# The Relationship between Levels of Physical Activity and Academic Achievement among Medical and Health Sciences Students at Cyberjaya University College of Medical Sciences

**DOI:** 10.21315/mjms2018.25.5.9

**Published:** 2018-10-30

**Authors:** Q-En Chung, Surajudeen Abiola Abdulrahman, Mohamad Khan Jamal Khan, Hassan Basri Jahubar Sathik, Abdul Rashid

**Affiliations:** 1 Department of Public Health Medicine, Penang Medical College, 10450 Georgetown, Pulau Pinang, Malaysia; 2 Faculty of Occupational Safety and Health, Cyberjaya University College of Medical Sciences, 63000 Cyberjaya, Selangor, Malaysia; 3 Centre of Collegiate Programmes, Cyberjaya University College of Medical Sciences, 63000 Cyberjaya, Selangor, Malaysia

**Keywords:** prevalence, physical activity, academic achievement, medical and health sciences, undergraduate students

## Abstract

**Background:**

This study aimed to explore the prevalence of physical activity among medical and health sciences students at Cyberjaya University College of Medical Sciences (CUCMS) and to determine the relationship of their physical activity level with their academic achievement and self-determination level.

**Methods:**

An analytical cross-sectional study was conducted among 244 Medical and Health Sciences undergraduate students at CUCMS from January to April 2017 using self-administered short-form version of the International Physical Activity Questionnaire (IPAQ-SF) and the third version of the Behavioural Regulation in Exercise Questionnaire (BREQ-3). Multiple regression models were fitted using SPSS version 20 to examine the relationships between study variables.

**Results:**

Half of the male students (51.7%) were in the health-enhancing physical activity (HEPA) group, as compared to only 24.7% of females. The odds of having a good grade point average was twice as high among HEPA active students (odds ratio [OR] = 1.89, 95% CI [1.09, 3.27], *P* = 0.023) than among non-HEPA active students. Further, the odds of being HEPA active was higher for males (OR = 3.16, 95% CI [1.61, 6.14], *P* < 0.01) than for females and higher for overweight students than for normal weight students (OR = 2.58, 95% CI [1.24, 5.57], *P* = 0.017). The odds of being HEPA active was 1.79 times higher for each unit increase in the integrated regulation score (OR = 1.79, 95% CI [1.14, 2.91], *P* = 0.020).

**Conclusion:**

The prevalence of physical inactivity was higher among females than males. This study also confirmed a significant association between physical activity level and academic achievement. HEPA active students performed better academically than those who were non-HEPA active.

## Introduction

Physical inactivity, besides being a key risk factor for non-communicable diseases, is one of the leading risk factors for death worldwide and contributes to approximately 3.2 million annual deaths globally. Therefore, the World Health Organization (1) recommends that adults aged 18 to 64 years engage in moderate intensity physical activity 5 days per week for at least 30 min per session.

Regular participation in physical activity has positive effects on physical, social, and mental health (2, 3). It reduces the risk of developing chronic diseases, enhances the quality of life and well-being, and improves cognitive functioning (4–7). Despite the evidence of these holistic benefits and ongoing governmental efforts to promote health, many still do not achieve the recommended level of physical activity to gain the potential benefits. The National Health and Morbidity Survey (NHMS) (8) estimated about 75% of adults in Malaysia were insufficiently active in the year 2015. This may be explained by a lack of motivation (9) to engage in regular physical activity at both population and individual level.

Generally, health professionals are believed to have a better understanding of the benefits of regular physical activity. They are also more likely to be effective (10–12) in providing counselling on health-related changes in behaviour if they practise healthy habits themselves. The health status of health professionals has been shown to influence their confidence level when counselling patients on physical activity. Individuals who are within the normal range of body mass index (BMI) and have achieved the vigorous physical activity levels described by the United States Department of Health and Human Services guidelines (13) have been shown to be more confident in health promotion counselling than those who are overweight or obese (11).

The exact nature of the relationship between physical activity levels and academic achievement is poorly understood. Most studies have suggested positive relationships (14–26), while some have suggested negative (27, 28) and non-significant associations (29). Even when significant associations have been found, the level of physical activity, to what extent it predicts good academic achievement, and the minimum level of physical activity required to achieve better grades have not been clarified. In addition, prevalence studies conducted among medical and health sciences students in Malaysia are limited.

This paper aimed to explore the health status (based upon the prevalence of physical activity level and nutritional status) among undergraduate medical and health sciences students at Cyberjaya University College of Medical Sciences (CUCMS) and to determine the relationship of this health status to their academic achievement and self-determination level.

## Materials and Methods

### Study Location and Context

This study was conducted among undergraduate students of CUCMS, which is located in Cyberjaya in the state of Selangor, Malaysia. This university was established in 2005 and offers a wide range of medical and health sciences programmes for undergraduates and has an approximate student population of 2,500. The bachelor programmes include medicine, pharmacy, psychology, physiology, biomedical engineering, occupational safety and health (OSH), and homeopathic medical science (HMS).

### Study Design

The current study was an analytical cross-sectional study conducted among 276 full-time undergraduate CUCMS students from Malaysia aged between 18 and 38 years. Students were chosen via a multistage cluster random sampling method. Four of the seven bachelor’s degree programmes were first selected by random drawing: medicine, pharmacy, OSH, and HMS. Next, the classes to participate were randomly selected from each programme by choosing from classes whose students were attending from the second semester onwards and were in their pre-clinical studies. All the students in each selected class were included in the study so that the required student numbers were proportionate to the number of students in each programme. To be included in this study, participants were required to be Malaysians aged between 18 and 38 years old and enrolled as full-time undergraduate students in the seven programmes stated above. We excluded students in their first semester or in the clinical phase of their programme during the study period as well as those who had sustained an injury that affected their overall physical activity level during the previous semester.

### Sample Size

We estimated the sample size for the study using Stata 13.0 software and a formula for a comparison between two proportions that a sample of 230 respondents would provide, with at least 80% power for detecting a difference of up to 6% lower prevalence of students with poor academic grades (as compared to 21% prevalence from a similar study by Nayak et al.) (18), assuming a two-tailed test and type 1 error rate of 5%. Taking into account a possible 20% non-response rate, the final sample size for the study was 276.

### Ethical Approval

This study was conducted in line with the Declarations of Helsinki, 2013 (30). Written approval for conducting this study was obtained from Penang Medical College Institutional Research Ethics Committee as well as from the CUCMS Research Ethics Committee prior to the commencement of the study. Written informed consent was obtained from all individual students enrolled in the study.

### Data Collection

The dean for each programme were met to explain the purpose of the study, its methodology, and to arrange for meetings with all six of the selected classes, which included one each from medicine and pharmacy, and two each from OSH and HMS. A total of six meetings were scheduled and these were held on six separate days between lectures at CUCMS. On a typical day of meeting the students, the lecturer-incharge briefed the students on the purpose of the meeting. Then, the researcher and research assistants took over the sessions to further clarify the purpose, benefits, and risks of the study and afterwards obtain the written informed consent from potential participants. Self-administered questionnaires were then distributed to consenting adult students after satisfactorily addressing their queries or concerns about their involvement in the study.

Data on sociodemographic profile (age, gender, race, bachelor programme), anthropometric measures (height and weight), health status (nutritional status, injury status, physical activity level, and average sitting time), and level of self-determination in engaging in physical activity among participants were retrieved and extracted onto standardised data extraction sheets from the pre-validated, self-administered questionnaires: the short form of the International Physical Activity Questionnaire (IPAQ-SF) and the third version of the Behavioural Regulation in Exercise Questionnaire (BREQ-3).

The IPAQ, suitable for population-based prevalence studies, was designed to obtain data on physical activity. It has gained global acceptance in many settings and has been translated into many languages since it was first introduced in 1998 (31). It has been validated for use in both developed and developing countries (32). The short version was used in this study due to its feasibility despite that the longer version provides more details on the various domains. Based upon a study conducted by Craig et al (32), the reliability between both versions has been shown to be comparable, with a demonstrated test-retest repeatability/reliability (Spearman’s *ρ*) of about 0.8. The criterion validity of IPAQ-SF was also reported to be comparable to most other self-report validation studies with a median of about 0.3 (32).

Self-determination level towards exercise was measured using the BREQ-3 (33, 34), which corresponds to different forms of behavioural regulation that lie on a continuum. Ranging from non-self-determined to completely self-determined, it is based upon the organismic integration theory, which was described by Ryan and Deci (35) as the extent to which an individual’s behaviour regulation is determined by the self or by external forces. The BREQ version 2 did not include the integrated regulation subscale; however, Markland and Tobin (33) demonstrated the excellent fit of this model. The Cronbach’s alpha for reliability for each dimension of BREQ-2 is above 0.70 (33). The instrument works well especially with the addition of the introjected regulation to the latest version, BREQ-3.

Official grade point average (GPA) records for the previous semester/year were obtained with permissions from the deans of the different programmes to replace self-reported GPAs as 60% of the respondents reported an unacceptable range of results during a randomised cross-check and validation of GPA scores based upon 10 observations. Although respondents were unaware that the official GPA records were obtained, the data was anonymised and observed discrepancies were not reported to the deans or students themselves and were solely used for analysis purposes. A total of 244 out of the 276 participants enrolled at study commencement were included in the final analysis. Six participants with injuries sustained in the previous semester and 14 participants with an incomplete data set were excluded from the final analysis. Data extraction forms were reviewed periodically for completeness, correctness, and accuracy.

### Data Analysis

To determine the physical activity level of each participant, a scoring protocol (32) for IPAQ-SF and a flow chart algorithm were used as guidelines for analysis. Metabolic equivalent task (MET) is used to express the intensity of physical activities. It is the ratio of the working metabolic rate relative to the resting metabolic rate. One MET is equivalent to a caloric consumption of 1 kcal/kg/h based on a 60 kg-person. Below shows the intensity of physical activity derived from 1 MET:

**Table:** 

Vigorous	8.0 MET
Moderate	4.0 MET
Walking	3.3 MET

A continuous score of physical activity level in the last 7 days for each participant was then obtained using the formula shown below based upon the derived intensity level:

$$\text{MET}-\text{min}/\text{week}=\text{MET}\times \text{minutes}\times \frac{\text{weight }(\text{kg})}{50\;\text{kg}}\times \text{number of days}$$

It is expressed in MET-minutes per week (MET-min/week), taking into account the frequency, intensity, and duration of the activities as well as body weight. These continuous scores were then categorised according to WHO classification (8) for physical activity levels: health-enhancing physical activity (HEPA) active, minimally active, or inactive. The HEPA active level of physical activity is equivalent to performing vigorous-intensity activity at least 3 days a week, achieving a minimum of 3 h per week (1,500 MET-min/week) or performing at least a light-intensity activity (e.g. walking) everyday, achieving a minimum of 15 h per week (3,000 MET-min/week). Performing at least a light-intensity activity for at least 5 days, achieving a minimum of 600 MET-min/week but less than 3000 MET-min/week warrants a classification of minimally active. Individuals who do not meet any criteria mentioned above fall under the inactive category. For ease of analysis and the purposes of this study, we further classified these categories into HEPA group and non-HEPA group (minimally active and inactive) since health and functional capacity benefit from being HEPA active only (8).

The BREQ-3 is the latest revised version of this questionnaire and consists of 24 items that measure self-determination levels towards exercise for six dimensions of external motivation. These dimensions are addressed along a continuum of internalisation based upon the organismic integration theory of self-determination theory (SDT), including amotivation, external regulation, introjected regulation, identified regulation, integrated regulation, and intrinsic regulation. Responses are indicated using a 5-point Likert scale showing agreement with different statements, for example ‘It’s important to me to exercise regularly’ and ‘I exercise because it’s fun’, reflecting underlying reasons to engage or not to engage in physical activity. Multidimensional scoring by calculating mean scores for each set of items in each dimensions was used here instead of relative autonomy index (RAI), which is no longer recommended as there are statistical arguments against its use (36).

We applied the standard WHO BMI range of scores to classify each respondent’s nutritional status into four categories. Similarly, we classified each respondent’s GPA score as ‘good’ or ‘poor’ using a cut-off point of 3, which is in line with the standards for good academic standing among most undergraduate programmes (37, 38) approved by the Malaysian Ministry of Higher Education (MOHE) with Malaysian Qualification Agency (MQA) accreditation (39). Good academic standing is usually set by a university or any higher learning authority recognised by the Malaysian Senate and is given preference for postgraduate programme applications (40, 41). Multiple regression models were used to examine the relationships between physical activity level, GPA score, and BREQ-3 dimensions and their predictors using SPSS version 20. Statistical significance was set at *P* < 0.05.

## Results

### Distribution of Respondents’ Sociodemographic Variables, Anthropometric Measures, Health Status, Academic Achievement, and Self-Determination Level

The mean age of the participants was 21.3 years old (SD = 1.8; 95% CI [21.1, 21.5]), with three-quarters of them being female (76.2%). The majority were of Malay ethnic, accounting for 93.4% (228/244) of the study population. About half of the participants were studying pharmacy (*n* = 119, 48.8%), 36.1% studying medicine (*n* = 88), and 7.4% and 7.8% studying OSH (*n* = 18) and HMS (*n* = 19), respectively. Five participants (2%) reported having sustained minor injuries during the previous semester, but the injuries did not affect their physical activity level, and so they were included in the study. As for self-determination level, higher means were observed for identified regulation, integrated regulation, and intrinsic regulation. Identified regulation ranked the highest among the BREQ-3 dimensions. These are shown in Table 1.

In Table 2, it is observed that males were generally and significantly taller and heavier than females (*P* < 0.01). The mean daily sitting time for females (561 min/day) was about an hour more than males (510 min/day). Of the population, 5.2% (*n* = 3) of the males were underweight compared to 17.2% (*n* = 32) of the females. The majority (about 65%) of the respondents of either gender (male = 38, female = 120) had a BMI within the normal range. It was observed that a twice as high proportion of males (*n* = 30, 51.7%) than females (*n* = 46, 24.7%) were HEPA active (*P* < 0.01).

The GPA scores were first categorised into four levels. They ranged from 1.9 to 4.0 with a 1% failure rate. These GPA scores were then categorised into two levels of good (3.00–4.00) and poor (< 3.00) for statistical analysis. Among them, 62.1% (*n* = 36) of males had poor grades as compared to 44.1% (*n* = 82) of females (Table 2). The observed difference was statistically significant (*P* = 0.017).

### Association of Sociodemographic Variables, Health Status, Academic Achievement, and Self-Determination Level with Physical Activity Levels

Univariable (simple) and multivariable (multiple) binary logistic regression analyses were conducted to test the association between independent predictor and outcomes (physical activity level and GPA scores). The regression analysis shown in Table 3 revealed that the odds of achieving a good GPA score was about twice as high for those who were HEPA active (odds ratio [OR] = 1.89; 95% CI [1.09, 3.27]; *P* = 0.023) than for non-HEPA active. The model explained 3% of variance in academic achievement (Nagelkerke *R*^2^ = 0.03).

Table 4 shows a summary of simple and multiple logistic regression analyses of the sociodemographic variables and health status on physical activity level. Hosmer-Lemeshow test showed that all variables but race were a good fit for selection into multiple logistic regression (*P* < 0.25) model. This final regression model was statistically significant (*χ*^2^ = 26.5, df = 8, *P* < 0.01) and predicted 74% of the outcome categories correctly. It explained 15% of the variance in physical activity level (Nagelkerke *R*^2^ = 0.15). It was observed that the odds of being HEPA active among males was three times higher than for females (OR = 3.16, 95% CI [1.61, 6.14], *P* < 0.01), whereas overweight respondents had an approximate 2.6 times higher odds (OR = 2.58, 95% CI: [1.24, 5.57], *P* = 0.017) of being HEPA active than individuals within the normal range of BMI.

Table 5 shows the results of the multiple logistic regression analysis that was conducted to examine if the self-determination level measured by the BREQ-3 dimensions predicted physical activity level. Following simple logistic regression analyses of an association between each BREQ-3 dimension and physical activity level, all BREQ-3 dimensions but external regulation qualified for selection into the multiple logistic regression model based upon a Hosmer-Lemeshow criteria of *P* < 0.25. The final regression model was statistically significant (*χ*^2^ = 28.8, df = 5, *P* < 0.01) and predicted 68% of the outcome categories correctly. It explained 16% of variance in physical activity level (Nagelkerke *R*^2^ = 0.16). It was observed that the odds of being HEPA active was 1.8 times higher for every unit increase in integrated regulation score (OR = 1.79, 95% CI [1.14, 2.91], *P* = 0.020).

### Association between Sociodemographic Variables, Nutritional Status, and Self-Determination Level

Table 6 illustrates the statistically significant variables obtained from the preliminary univariable (simple) linear regression analysis of the sociodemographic, anthropometric, and health status variables with each BREQ-3 dimensions. Using the ‘enter’ method of variable selection, all significant predictors at univariable level were included in the multiple linear regression model. It was observed that for each unit increase in age, amotivation score decreased significantly by 0.06 units (B = −0.06, *P* = 0.011), and females had a lower amotivation score by 0.32 units as compared to males (B = −0.32, *P* < 0.01). Lastly, for every unit increase in weight (kg), external regulation score increased by 0.012 units (B = 0.012, *P* < 0.01). In each univariable analysis and the final multivariable model, the independent predictor variables explained a minimum of 5% variance in the outcome variable.

## Discussion

### Study Population

This study population consisted of 244 undergraduate students that are currently studying medicine, pharmacy, OSH, and HMS at CUCMS. The average age of the study population was 21 years old and majority of them were ethnic Malay (93.4%).

### Prevalence of Physical Activity Level

The findings of this study revealed that 51.7% of males and 24.7% of females met the WHO recommendation of physical activity (HEPA active), which, although slightly higher, is comparable with male-female distribution estimates from the 2015 NHMS report, where the prevalence of physical activity (HEPA active) among males and females was reported to be 34% and 16%, respectively. A few other studies have also shown that men were significantly more active than women (42–45). Males were more likely to engage in sports and exercise that were higher in intensity, whereas females preferred daily physical activity such as walking and biking (46). It has been suggested that the difference in physical activity participation and performance between genders is influenced by sexual stereotypes and gender roles (47) internalised since childhood. In line with social role theory and social asymmetry between genders, masculinity is perceived as the norm in sports even among female athletes and in sports perceived as feminine (48).

### Prevalence of Nutritional Status

About 35% of students from each gender had a BMI out of the normal range. More females (17.2%) than males (5.2%) were underweight, and more males were overweight (20.7%) and obese (8.6%) than females (14% and 4.3%, respectively). This is consistent with a recent study performed among students at another medical school in Malaysia (49) that revealed a similar distribution in nutritional status: 18% of males and 14% of females were overweight; 9% of males and 2% of females were obese.

### Physical Activity Level and Academic Achievement

This study produced results that corroborate the findings of much of the previous work in this field. Our study confirms that physical activity level is associated with academic achievement which is consistent with recent studies and systematic reviews that have observed significant positive associations among medical students and other college and elementary school students in different parts of the world (14–26). We observed that the odds of having good GPA scores (3.00–4.00) was twice as high among HEPA active students than among non-HEPA active students. This suggests that the minimum level of physical activity required to achieve better academic grades is HEPA active.

It can thus be suggested that hypothetical theories on the mechanism of physical activity providing cognitive changes are correct. The positive effects of physical activity are not limited to improved blood circulation to the brain (50), the growth of new brain cells, blood vessels, and protein brain-derived neurotrophic factor (BDNF) (51, 52), and increased levels of neurotrophins and neurotransmitters, but a combination of all these factors along with changes in synaptic plasticity (53) and spine density that have the potential to mediate the beneficial effects on learning and memory.

### Physical Activity Level and Self-determination Level

The BREQ-3 questionnaire is based upon the organismic integration theory of SDT. It comprises both intrinsic and extrinsic components, which in this case are dimensions of motivation that lie along a continuum. Amotivation refers to a person who exercises without feeling any motivation, while intrinsic regulation is very much autonomous (54), being a situation where people run because they like running and feel good by doing so. External and introjected regulation are considered in the spectrum of lower self-determination to exercise. The main motivator found in these two types is either a desire to obtain rewards or avoid punishment (55). The difference lies between the types of rewards or punishment; either they are of external or intrinsic nature. A person with external regulation exercises to please other people, while someone with introjected regulation exercises for intrinsic factors, such as ego, or avoiding self-inflicted punishments such as guilt or shame. Identified and integrated regulation are autonomous forms of motivation (55). Identified regulation is significant at a personal level and the outcomes are valued by the individual. For example, this means that a person would perform cardiovascular exercises for better heart function. Integrated regulation, however, is a belief that one’s behaviour projects one’s identity, and is consistent with personal values (55). People who demonstrate this motivation would go cycling believing that they are cyclists, and hence, cycling is consistent with their identity.

Our results showed that integrated regulation is the only one of the BREQ-3 dimensions that significantly predicts physical activity level. The odds of being HEPA active increases by 1.8 times for every unit increase in integrated regulation score. In other words, the findings suggest that Medical and Health Sciences students at CUCMS were mainly motivated by their own personal beliefs, and they perhaps identified with the belief that they are future healthcare workforce, and so they engaged in regular physical activity to maintain health for this reason. Participants may also have identified as students, in which case peer pressure, consciousness of body image, and access to social media might have influenced their behaviour towards exercise. Thus, it would be the consequences, or in this case, the reward of having a better body image that the participants would have liked to obtain rather than the pure enjoyment of exercise, which would be fully intrinsic. This finding also agrees with a previous study (56) on measuring integrated regulation. The current study has confirmed that integrated regulation is the only significant independent predictor of exercise behaviour, which is also consistent with findings of a previous study (57). Not only is this measure valid for this construct, it also increases the fidelity of measurement instruments of SDT. This discovery had been shown to have a great potential for developing interventions that apply the concept of integrated regulation to the improvement of self-determination levels for increasing physical activity levels (58, 59).

### Self-reported GPA versus Official GPA Records

We used official GPA records for data analysis rather than the self-reported GPAs. A random cross-checking and validation of self-reported versus official GPA records was performed on ten respondents and it was found that six of them reported results that were significantly higher than the actual results. Additionally, about 12% (17/144) of the self-reported GPA data were missing. The mean of the self-reported GPA scores was 3.17 whereas that of official GPA record was 3.06. This showed a mean difference of 0.11. The difference in the accuracy of the self-reported GPAs could have been due to factors of recall bias and social desirability. About a quarter more students (75.4% versus 51.6%) with an official GPA record of less than 3.00 reported GPA scores of 3.00–4.00. This is consistent with a study done by Kuncel et al (60), in which the authors observed that students with good grades reflected reliable self-reported results, whereas poor grades students exhibited greater inflation of GPA score.

Obtaining official GPA records would be the ideal condition; however, there are circumstances that may limit access, for instance confidentiality and administrative procedures and regulations. Only under these circumstances would the researcher need to compromise and rely upon self-reported GPA scores. It is possible that a more private than public (i.e. among peers) location for questionnaire administration and completion would have ensured better anonymity and privacy and therefore improved the reliability of the self-reported GPA scores.

### Strengths

To the best of our knowledge, this is the first epidemiological study to report the prevalence and association between physical activity, academic achievement, and self-determination levels among undergraduate medical and health sciences students in Malaysia. Given the strategic importance of this pool to the future healthcare workforce, it is important not only to encourage physical activity amongst them for the benefit of superior academic performance, but ultimately also to encourage the recommended level of physical activity as a primary preventive approach for non-communicable diseases that could directly impact the economy and health care system. The use of validated tools (IPAQ-SF and BREQ-3) not only ensured the internal validity of our estimates but also assured comparability with international studies. GPA results were cross-checked with official results and this increased the validity and reliability of the study results.

### Limitations

The difference between our prevalence estimates and those reported by the NHMS data could be due to several factors. With self-reported data, respondents might have overestimated their physical activity level and hence subject the information not only to social desirability bias but also to misclassification bias (61). Misclassification bias could also have occurred when reporting the amount of time spent according to the intensity of physical activity. Although examples of physical activity were listed in the questionnaire, some common ones such as climbing stairs, were not included in any of the physical activity intensity questions. Social desirability amongst the overweight respondents may have influenced the degree of over-reporting of physical activity level (62, 63) amongst them, which could have influenced our results towards its current direction.

Other limitations of this study include the inability to establish causal/temporal relationship between physical activity and academic achievement due to the study’s analytical cross-sectional design. In addition, self-administered questionnaires are limited by social desirability bias, recall bias, inaccurate and incomplete data, and questionnaire fatigue. Lastly, given its limited scope, certain confounding factors that were not tested in this study may have influenced the findings. These include social factors such as time management and interpersonal interaction, environmental factors such as conducive learning environment, psychological factors such as depression, anxiety, and stress level, and physiological factors such as underlying medical condition and fatigue. We suggest that future longitudinal studies incorporate these potential confounders into their study design so as to isolate their potential contributory nature to the overall association between physical activity and academic performance.

## Conclusion

The prevalence of the WHO recommended level of physical activity (HEPA active) among undergraduate Medical and Health Sciences students in this study was 52% and 25% among males and females, respectively. This study confirms a significant positive association between physical activity level and academic achievement. The odds of achieving good GPA score was twice as high among those who were HEPA active than those who were non-HEPA active. Integrated regulation was the only significant predictor of physical activity level among the BREQ-3 dimensions. Interventions incorporating this measure have a great potential to influence the motivation for physical activity.

## Figures and Tables

**Table 1:** Distribution of respondents’ sociodemographic variables, health status, and self-determination level in engaging physical activity

Variables	Mean (SD)	95% CI
Age	21.3 (1.8)	21.1, 21.5
BREQ-3 Dimensions		
Amotivation	0.55 (0.67)	0.46, 0.64
External Regulation	1.20 (0.87)	1.09, 1.31
Introjected Regulation	2.02 (0.92)	1.90, 2.14
Identified Regulation	2.88 (0.67)	2.80, 2.96
Integrated Regulation	2.11 (0.96)	1.99, 2.23
Intrinsic Regulation	2.77 (0.79)	2.67, 2.87
	
	***N* (%)**	
	
Gender		
Male	58 (23.8)	
Female	186 (76.2)	
Race		
Malay	228 (93.4)	
Non-Malay	16 (6.6)	
Programme		
Medicine	88 (36.1)	
Pharmacy	119 (48.8)	
OSH	18 (7.4)	
HMS	19 (7.8)	
Injury Status		
Yes	5 (2.0)	
No	239 (98.0)	

Abbreviation- BREQ-3: Behavioural Regulation in Exercise Questionnaire-version 3. OSH: Occupational Safety and Health. HMS: Homeopathic Medical Science

**Table 2:** Distribution of respondents’ anthropometric measures, health status, and academic achievement by gender

Variables	Gender				*P*-value

	Male (*n* = 58)		Female (*n* = 186)		
	
	Mean (SD)	95% CI	Mean (SD)	95% CI	
Height (cm)	170.4 (6.9)	168.6, 172.2	157.6 (6.6)	156.7, 158.6	< 0.01*^a^
Weight (kg)	68.5 (13.9)	64.8, 72.1	54.9 (11.4)	53.3, 56.6	< 0.01*^a^
Sitting (min/day)	510 (201)	457, 562	561 (259)	524, 599	0.114^a^
		
	***N* (%)**		***N* (%)**		
		
Nutritional Status					
Underweight	3 (5.2)		32 (17.2)		0.063^b^
Normal	38 (65.5)		120 (64.5)		
Overweight	12 (20.7)		26 (14.0)		
Obese	5 (8.6)		8 (4.3)		
Physical Activity Level					
Non-HEPA	28 (48.3)		140 (75.3)		< 0.01*^b^
HEPA	30 (51.7)		46 (24.7)		
GPA					
Poor (< 3.00)	36 (62.1)		82 (44.1)		0.017*^b^
Good (3.00–4.00)	22 (37.9)		104 (55.9)		

* Significant at *P* < 0.05

a *P*-value obtained from student *t*-test for independent samples

b *P*-value obtained from chi-square test

Abbreviation- HEPA: Health-enhancing physical activity. GPA: grade-point average

**Table 3:** Binary logistic regression analysis of physical activity level predicting GPA scores (*N* = 244)

Variables	β	OR	95% CI	*P*-value
Physical Activity Level				
Non-HEPA	Reference	1.00		
HEPA	0.64	1.89	1.09, 3.27	0.023*

* Significant at *P* < 0.05. Reference category is good GPA scores (3.00–4.00)

Abbreviation- HEPA: Health-enhancing physical activity. OR: odds ratio

**Table 4:** Multiple logistic regression analysis showing association between sociodemographic variables, health status, and physical activity level (*N* = 244)

Variables	β	Crude OR†	Adjusted OR†† (95% CI)	*P*-value
Gender				
Female	Reference			
Male	1.15	3.26	3.16 (1.61, 6.14)	< 0.01*
Programme				
HMS	Reference			
Medicine	1.03	2.36	2.80 (0.73, 11.12)	0.141
Pharmacy	1.18	2.60	3.25 (0.82, 12.49)	0.086
OSH	0.82	3.39	2.28 (0.44, 11.78)	0.324
Nutritional Status				
Normal	Reference			
Underweight	−0.54	0.49	0.58 (0.23, 1.53)	0.276
Overweight	0.95	2.36	2.58 (1.24, 5.57)	0.017*
Obese	0.004	1.05	1.00 (0.31, 3.74)	0.995
Injury Status				
No	Reference			
Yes	1.05	3.41	2.86 (0.42, 21.04)	0.303

* Significant at *P* < 0.05. Reference category is HEPA active

† Odds ratio obtained from simple logistic regression

†† Odds ratio obtained from multiple logistic regression model

Abbreviation- HEPA: Health-enhancing physical activity. OSH: Occupational Safety and Health. HMS: Homeopathic Medical Science. OR: odds ratio

**Table 5:** Multiple logistic regression analysis showing association between self-determination level and physical activity level (*N* = 244)

Variables	β	Crude OR†	Adjusted OR†† (95% CI)	*P*-value
BREQ-3 Dimensions				
Amotivation	−0.32	0.64	0.73 (0.53, 1.22)	0.183
Introjected Regulation	−0.27	1.41	0.76 (0.54, 1.24)	0.229
Identified Regulation	0.14	2.19	1.15 (0.65, 2.29)	0.681
Integrated Regulation	0.58	2.02	1.79 (1.14, 2.91)	0.020*
Intrinsic Regulation	0.43	2.37	1.54 (0.92, 2.73)	0.124

* Significant at *P* < 0.05. Reference category is HEPA active

† Odds ratio obtained from simple logistic regression

†† Odds ratio obtained from multiple logistic regression model

Abbreviation- OR: odds ratio. BREQ-3: Behavioural Regulation in Exercise Questionnaire-version 3

**Table 6:** Multiple linear regression of sociodemographic characteristics and health status on self-determination level (*N* = 244)

Variables	B	SE	*t*	95% CI	*P*-value
Amotivation					
Age	−0.06	0.02	−2.57	−0.11, −0.01	0.011*
Gender	−0.32	0.1	−3.25	−0.52, −0.13	< 0.01^**^
External Regulation					
Gender	−0.22	0.14	−1.53	−0.49, 0.06	0.13
Weight	0.012	0.005	2.69	0.003, 0.021	0.01*
Nutritional Status	0.002	0.05	0.03	−0.09, 0.09	0.97
Introjected Regulation					
Height	0.008	0.008	0.92	−0.009, 0.02	0.36
Weight	0.009	0.005	1.67	−0.002, 0.02	0.10
Integrated Regulation					
Gender	−0.23	0.18	−1.27	−0.59, 0.13	0.21
Height	0.018	0.01	1.96	0.00, 0.036	0.05

* Significant at *P* < 0.05
